# Advances in Targeted Therapy for the Treatment of Cervical Cancer

**DOI:** 10.3390/jcm12185992

**Published:** 2023-09-15

**Authors:** Dean E. Watkins, Daniel J. Craig, Shahnaz D. Vellani, Ahmad Hegazi, Kaylee J. Fredrickson, Adam Walter, Laura Stanbery, John Nemunaitis

**Affiliations:** 1 University of Toledo Medical Center, College of Medicine and Life Sciences, University of Toledo, Toledo, OH 43614, USA; 2 ProMedica Toledo Hospital, Toledo, OH 43606, USA; 3 Gradalis, Inc., Dallas, TX 75006, USA

**Keywords:** cervical cancer, immunotherapy, HPV, checkpoint inhibitor

## Abstract

Cervical cancer is an international public health crisis, affecting several hundred thousand women annually. While not universally protective due to other risk factors, many such cases are preventable with vaccination against high-risk serotypes of the human papilloma virus (HPV 6, 11, 16, 18, 31, 33, 45, 53, 58). Advanced-stage and recurrent cervical cancers are typically lethal and have been the focus in recent years of the integration of immune checkpoint inhibitors (CPIs) to improve survival. We have consolidated information regarding the role of the immune system in both disease progression and disease clearance with the aid of targeted therapies and immunotherapeutic agents. Additionally, we have characterized the treatment modalities currently indicated as the standard of care—such as bevacizumab and the immune CPIs—and those recently approved or in development, including Tivdak, Vigil, and chimeric antigen receptor (CAR) T-cells.

## 1. Introduction

Cervical cancer is the fourth most commonly diagnosed cancer in women worldwide, with more than 569,000 cases and approximately 311,000 disease-related deaths in 2018 according to the World Health Organization (WHO). Internationally, cervical cancer is the second leading cause of cancer mortality in women aged 20–39 and continues to be responsible for more than eight million disability adjusted life years lost annually [1,2,3]. More than 85% of cervical cancer mortalities occur across 43 underdeveloped nations, including Malawi and Zimbabwe, which see a five-year survival rate of less than 30%, contribute to a mortality rate 18 times greater than that in the most developed countries [4,5,6,7] According to the Surveillance, Epidemiology, and End Results (SEER) database, women in the United States (US) with cervical cancer had a 66.3% five-year survival rate from 2011 to 2017 [8]. In the US, the incidence of and mortality from cervical cancer have steadily declined since the 1970s due to the wide-scale implementation of cytological screening programs using the Papanicolaou test (Pap smear). Significant risk factors include a history of human papilloma virus (HPV) infection, early sexual debut, multiple sex partners, high-risk sexual behavior, immunosuppression, sexually transmitted infection (STI) history, vulvar or vaginal dysplasia, and a history of tobacco smoking. Smoking status, duration, and amount smoked are associated with double the risk of high-grade lower genital tract dysplasia after adjusting for HPV status, while smoking cessation was shown to result in a two-fold risk reduction. Additionally, lack of screening is a significant risk factor [9]. The reduction in cervical cancer mortality in the US is due to greater awareness of these cervical cancer risk factors, the aforementioned increased accessibility to screening techniques, and last, improved treatment and prevention plans [4]. For example, countries such as the US, with a 50% vaccination rate in eligible women and girls, were shown to have a nearly 70% reduction in HPV 16 and 18 infections [9].

According to the American College of Obstetricians and Gynecologists (ACOG) and the United States Preventative Services Taskforce (USPSTF) guidelines, women aged 21–29 are recommended to be screened via Pap smear cytology every three years. Women between 30 and 65 years old have the option to undergo cytology alone every three years, high-risk HPV (hrHPV) testing alone every five years, or combination cytology and hrHPV every five years. Screening after age 65 is not indicated for women who screened negative via cytology three times or had negative hrHPV/co-testing twice [10]. Women who have had a hysterectomy or trachelectomy are not indicated for screening as long as they do not have a history of high-grade cervical lesions or cancer [10]. Socioeconomic status has been shown to play a large role in screening success, as many women who are uninsured, members of minority groups, immigrants, those with limited education, and women who lack routine primary care are less likely to be screened [1,11]. Screening is of particular importance in the prevention of cervical cancer, as surgical intervention is only an effective treatment method for early-stage disease. Surgical treatment for cervical cancer is based on stage and ranges from conization and simple hysterectomy to radical hysterectomy or radical trachelectomy with pelvic lymphadenectomy. Beyond surgical interventions, chemoradiation is the standard of care for locally advanced cervical cancer (stage IB3-IVA), and it involves weekly cisplatin combined with external beam radiation therapy, followed by brachytherapy. In recent years, the main topic of interest has been treatments for advanced and advanced recurrent cervical cancers using combination chemotherapy regimens, along with immunotherapy. There have been several large, randomized, controlled trials that have examined combination chemotherapy modalities for treating metastatic or advanced recurrent cervical cancers in an effort to prolong patient survival without sacrificing quality of life—including GOG 204, GOG 240, and JGOG 0505.

The GOG 204 trial was a phase 3, randomized, controlled trial that sought to evaluate the efficacy of several cisplatin doublet combinations in advanced recurrent cervical cancer. These patients were randomized into either the reference arm with paclitaxel plus cisplatin (P + C) or treatment arms that included vinorelbine plus cisplatin (V + C), gemcitabine plus cisplatin (G + C), or topotecan plus cisplatin (T + C). The results demonstrated that not only was there no significant improvement in OS in any of the treatment arms compared to the reference arm, but the trends in relative risk, progression-free survival, and OS also suggested that the reference arm (P + C) was superior to the combination therapies in the treatment arm [12].

The GOG 240 trial was a 2 × 2 trial that compared two combination chemotherapy modalities (cisplatin + paclitaxel and topotecan + paclitaxel) with and without the angiogenesis inhibitor bevacizumab to treat recurrent/persistent/metastatic cervical cancer. The results showed that the regimens that included bevacizumab resulted in significant improvement in OS (16.8 versus 13.3 months (*p* = 0.0068)), suggesting that angiogenesis inhibitors may supplement chemotherapy in the treatment of advanced cervical cancer [13].

The JGOG 0505 trial compared carboplatin-based chemotherapy regimens to the standard cisplatin-based regimens in the treatment of metastatic or recurrent cervical cancer. Patients with a history of metastatic or recurrent cervical cancer treated with <1 platinum-containing therapy and no prior taxane therapy were randomly assigned to either the conventional paclitaxel + cisplatin (T + P) arm or the paclitaxel + carboplatin (T + C) arm. The results showed that the median OS was 18.3 months with T + P versus 17.5 with T + C (noninferiority *p* = 0.032), demonstrating that T + C is not inferior to T + P [14].

These studies showed minimal variance among several chemotherapy regimens in improving patient survival in cases of metastatic or advanced recurrent cervical cancer. Although the trials were small, the potential to improve patient longevity shown with the addition of immune checkpoint inhibitors (CPIs) to standard chemotherapy regimens indicated the need to investigate targeted therapies further. As time has progressed, the push for more potent and more tolerable treatment methods to further improve patient outcomes has put a spotlight on immunotherapeutic agents. The recent discussion of possible options has included: tisotumab vedotin (Tivdak), gemogenovatucel-T (Vigil), chimeric antigen receptor (CAR) T-cells, and targeted therapies, such as immune CPI, which have been the most explored.

In this review, we discuss both novel and currently approved targeted therapies and immunotherapeutic agents for the treatment of advanced and recurrent cervical cancers that may be effective if preventive management with or without early-stage therapies fails or is not utilized.

## 2. Human Papilloma Virus Infection and Cervical Cancer Oncogenesis

HPV is among the most common cancer-causing infectious agents worldwide; however, most infected individuals—approximately 65% of women and 75% of men—experience complete viral clearance within 12 months [15,16]. The effective clearance of HPV by women is largely due to the host immune response, which begins at the time of initial HPV infection of keratinocytes at the basal layer of the cervical epithelium [17].

Keratinocytes, once infected with HPV, function as non-professional antigen-presenting cells via their Toll-like receptors (TLRs), both at their cell surface and within the endosomes. Activation of these TLRs, most notably endosomal TLR-9, stimulates production of many pro-inflammatory cytokines, such as TNF-α and type 1 interferons (IFNs). These cytokines lead to recruitment and upregulation of LCs and dendritic cells (DCs), respectively [18,19].

Stimulation of DCs and LCs is the first instance of professional antigen-presenting cell (APC) recruitment to the site of infection, which allows for the beginning of T-cell-mediated adaptive immunity. While both DCs and LCs are APCs, there are a few key distinctions in their functions due to differences in their TLR expression. While LC activation leads to production of the cytokine IL-15 and subsequent promotion of CD8+ T-cells, DC activation leads to a much wider array of cytokine production—IL-1β, IL-6, IL-8, IL-10, IL-12, GM-CSF, monocyte chemoattractant protein (MCP), and TGF-β. Notable cytokines produced in this array are IL-6 and IL-12, which are necessary for naïve B-cell activation and subsequent antibody production/class switching by B-cells to generate humoral immunity [20].

Even through the robust and amplified host immune response, many HPV subtypes have several methods of evading host immune recognition. These evasion methods are categorized as passive immune evasion strategies or aggressive immune evasion strategies [21]. A key passive immune evasion strategy employed by HPVs is a dynamic protein expression model—one that increases as the infection persists. During the early stages of infection in the basal layer of stratified squamous epithelium, the viral genome is quickly shuttled to the nucleus, and viral DNA replication is kept low due to the regulatory effects of viral proteins E1 (a helicase) and E2 (E6/E7 repressor protein) [4,21]. This process helps the virus to prevent detection by reducing the rate of antigen presentation to the host immune system. Once the infected basal cell exits the cell cycle and attempts to enter the differentiation stage, HPV activates and increases its gene expression through the continued activity of E1, as well as the activity of unrepressed E6 and E7, which delay cell differentiation and permit high-output viral replication [4,21]. As the infection progresses to later stages, protein expression rises, but due to the poor expression of antigen-presenting cells in the outer epithelial layer, most of the proteins are still not presented to the host immune system, instead being shed from the outer layer [21,22]. Some virus particles are able to infect neighboring cells at the time of shedding, permitting further propagation of the virus [4]. With persistent infection, there is an increased risk for the HPV genome to integrate into the host cell genome. Viral genome integration is a significant contributor to HPV-mediated tumorigenesis because it has been shown that the process regularly disrupts the gene encoding the E2 protein, thus leading to completely unregulated E6 and E7 activity [4].

The aggressive immune evasion strategy and oncogenicity expressed by high-risk HPV serotypes is derived from the virus-specific oncoproteins E6 and E7. Despite being found in all variants of HPV, these proteins are only essential for oncogenesis for the high-risk subtypes, such as HPV 16 and 18. Notably, E6 in such high-risk subtypes contains a PDZ-binding motif at its C-terminal domain, allowing it to bind to tumor proteins (p53) and ultimately causing it to be degraded [4]. This process is significant, as p53 is a critical tumor suppressor protein that leads to significantly increased cell dysregulation and cervical cancer progression when its expression is reduced [23,24]. Alternatively, conserved region 3 (CR3) of E7 functions as a zinc finger domain at the C-terminal end of the protein. This motif is of particular importance, as it is responsible for the inhibitory interactions between E7 and host proteins that are involved in both cell cycle regulation and apoptosis—specifically of p21 and pRb [4]. In high-risk subtypes of HPV, E7 specifically disrupts Rb binding to the E2F transcription factor, ultimately leading to the continuous promotion of the S-phase in host cells [21].

Overall, 70% of cervical cancer cases can be attributed to hrHPV strains 16 and 18. Subunit HPV vaccines provide recipients with resistance to both common and clinically significant serotypes of the virus. This series of vaccines specifically target the most prevalent low-risk (HPV 6, 11) and high-risk (HPV 16, 18, 31, 33, 45, 53, 58) HPV subtypes via injection of virus-like particles (VLPs) that mimic each HPV subtype being targeted [25]. While there are currently three FDA-approved HPV vaccines, the only one currently used in the US is Gardasil-9 (Merck), a nonavalent vaccine that targets all nine of the previously mentioned most prolific or high-risk HPV subtypes [26]. This vaccine has been shown to be 97% effective against these strains. The HPV vaccine is recommended for children beginning at nine years old, with catch-up vaccinations through age 26 for unvaccinated women. Women aged 27–45 may still be eligible for the vaccine and should engaged in shared decision making with their primary care physician [1,26]. Thus, HPV vaccination is an effective preventative method to minimize the risk of cervical cancer from HPV infection. Despite this encouraging information, vaccination rates among eligible, at-risk women in the United States remain unsatisfactory, lagging behind other developed countries. In 2015, only 43% of US women in their 30s were maintaining adequate screening via Pap smear. In 2017, 48.6% of US adolescents were up to date with the vaccination series [1]. Thankfully, vaccination rates have improved slightly in recent years, showing 53.7% of girls aged 13–17 and 53.6% of women aged 18–26 in the US having received the HPV vaccine by 2020 [11]. This increase is being credited to strong provider recommendations and increased patient awareness about HPV. However, disparities still exist between vaccination rates in Hispanic and Black women compared to White women aged 19–26 in the US, requiring further discussion regarding social determinants of health among these groups to promote improved national vaccination rates [11].

## 3. Angiogenesis Inhibitor

The anti-VEGF monoclonal antibody bevacizumab has been demonstrated to be effective as an anti-angiogenic therapy in both early- and late-stage cervical cancer cases [13,27]. Bevacizumab functions by binding to extracellular vascular endothelial growth factor A (VEGF-A). This action sequesters the growth factor from its receptor, vascular endothelial growth factor receptor (VEGFR), on endothelial cells. In the context of cervical cancer, this process is significant because cervical cancer cells have been found to particularly overproduce VEGF, resulting in the proliferation of local endothelial cells and a poorer prognosis due to increased tumor vascularity [28,29]. Thus, the application of bevacizumab as the standard immunotherapeutic in treating cervical cancer is unsurprising. In fact, when added to combinational chemotherapy with topotecan and paclitaxel, bevacizumab was found to significantly increase the median OS time in cervical cancer patients compared to those receiving cisplatin + paclitaxel chemotherapy alone (16.8 months vs. 13.3 months; *p* = 0.007) [13].

Despite its standard-setting efficacy and high tolerance in patients, bevacizumab therapy is associated with some risks—most commonly hypertension. Bevacizumab is thought to induce vasoconstriction by inhibiting nitric-oxide synthase and endothelial dysfunction, while also playing a role in inducing high blood pressure by reducing renal sodium excretion. Proteinuria from this renal damage has also been reported with bevacizumab therapy. Posterior reversible encephalopathy is a rare but reversible side effect of bevacizumab therapy; and it is associated with headaches, seizures, loss of vision, and emesis. Gastrointestinal (GI) perforations, recto-vaginal fistula formation, vesicovaginal fistula formation, non-GI fistula formation, hemorrhage, and thromboembolic events are rare but potentially fatal side effects of bevacizumab therapy [30,31]. Future consideration and examination of adjuvant therapies can further optimize care for patients with cervical cancer.

## 4. Anti-PD-1/PD-1L Monoclonal Antibodies

The programmed death-1 (PD-1) and programmed death-ligand 1 (PD-L1) axis has proven to be a valuable target in cancer therapy. Mechanistically, when the PD-1 receptor on T cells binds to PD-L1 on host cells, the inflammatory response typically initiated by T cells in response to an antigen is halted. This process is an evolutionary safeguard to protect against widespread autoimmune reactions. A variety of cancers use this mechanism to their advantage by over-expressing PD-L1 in an effort to prevent the immune system from effectively clearing the cancer cells [32].

Anti-PD-1 and anti-PD-1L monoclonal antibodies, better known as immune CPIs, disrupt the PD-1/PD-L1 interaction and result in a directed CD8+ CTL response against PD-L1-positive tumor cells [33]. This response has led physicians to measure the level of PD-L1 expression on cancer cells using immunohistochemistry to identify patients who may respond to anti PD-1/PD-L1 antibodies [34]. The improved efficacy proposed by utilizing either of these treatment methods stems from the finding that cervical cancer cells have increased PD-L1 expression, which assists these malignant cells in evading anti-tumor immune responses [35]. More interesting, however, is that the E7 protein from HPV16 appears to play a role in upregulating the expression of PD-L1, beyond its other oncogenic effects [36]. Thus, targeting this interaction by binding to either PD-L1 on the malignant cell or PD-1 on the CD8+ CTL should result in improved tumor clearance.

In the phase 2 KEYNOTE-158 study, pembrolizumab monotherapy (200 mg) was found to have an objective response rate of 14.6% (95% CI, 7.8–24.2%), with 83.7% of patients in the study (of 98 total patients) found to have PD-L1-positive tumors. Every patient who responded to pembrolizumab therapy in this study had PD-L1-positive tumors. For these patients, the median progression-free survival was 2.1 months (95% CI, 2.1–2.3 months). Additionally, the PD-L1 tumor population had an increase in median OS time (11 months vs. 9.4 months), as well as an increase in both six-month (80.2% vs. 75.2%) and 12-month (47.3% vs. 41.4%) estimated OS compared to the PD-L1-negative tumor population receiving pembrolizumab therapy [37]. Based on these results, it is clear that, while PD-L1 expression in malignant cervical tumors may result in a response to pembrolizumab therapy in some patients, there is a far greater likelihood of unresponsiveness when pembrolizumab is used as a stand-alone treatment method. Such reduced efficacy may be due to more clinically relevant responses to standalone pembrolizumab therapy requiring a toxic concentration of these anti-PD-1 antibodies, leading to global CD8+ CTL responses and subsequent inflammation. It is possible that combining anti-PD-1/PD-1L immune therapies with other immunotherapies or high-efficacy treatment methods could prove to be synergistic. However, the recent phase 2 clinical trial NCT02921269 used atezolizumab and bevacizumab in combination and did not support the presence of a synergistic effect. In this study, there was a confirmed Operational Readiness Review (ORR) of 0% and a median PFS of 2.9 months (95% CI, 1.8–6.0 months) [38]. An upcoming phase 3 clinical trial, CALLA, plans to examine the efficacy of durvalumab + chemoradiotherapy in patients with cervical cancer and seeks to provide insight into whether or not such a combinational therapy promotes a stronger immunogenic environment via increased cell death and antigen presentation [39].

The phase 3 KEYNOTE-826 trial investigated the use of pembrolizumab (200 mg) + paclitaxel (175 mg/m^2^) combination therapy with either cisplatin or carboplatin (at the preference of the investigator) compared to paclitaxel + platinum-based chemotherapy in patients with persistent, recurrent, or metastatic cervical cancer [40]. The experimental and control regimens were administered to participants once every three weeks for a total of six treatments. Of the 617 participants (308 experimental, 309 placebo), 63.6% of participants in the experimental group and 62.5% of participants in the placebo group received supplemental bevacizumab (15 mg/kg) at the discretion of the investigator [40]. Furthermore, on assessment of PD-L1 expression, 548 participants were found to have a PD-L1 combined positive score (the cumulative sum of PD-L1-positive cells detected in a sample divided by the total number of viable tumor cells in said sample, multiplied by 100) ≥ 1, and 317 participants were found to have a PD-L1 combined positive score ≥ 10 [40]. It was found that participants with a PD-L1 combined positive score ≥ 1 who received the regimen containing pembrolizumab had significantly longer median PFS than the control group (10.4 months vs. 8.2 months; HR = 0.62; 95% CI, 0.50–0.77; *p* < 0.001 [40]. Understandably, this significant improvement in median PFS was also seen in participants with a PD-L1 combined positive score ≥ 10 (10.4 months vs. 8.1 months; HR = 0.58; 95% CI, 0.44–0.77; *p* < 0.001) [40]. Additionally, based on a 24-month estimate, patients receiving pembrolizumab with a PD-L1 combined positive score ≥ 1 were found to have significantly longer OS than the control group (53% vs. 41.7%; HR = 0.64; 95% CI, 0.50–0.81; *p* < 0.001) [40]. Similarly, participants receiving pembrolizumab with a PD-L1 combined positive score ≥ 10 also saw a significant improvement in OS compared to the control group (54.4% vs. 44.6%; HR = 0.61; 95% CI, 0.44–0.84; *p* = 0.001) [40]. Last, while there appeared to be some benefit to bevacizumab co-administration, no significant improvement in treatment efficacy was observed compared to those who did not receive bevacizumab [40].

Pembrolizumab usage is not without its faults, however. Side effects associated with pembrolizumab immune therapy include nausea, fatigue, and anemia. Rare, but more severe, side effects noted during clinical trials included photosensitivity, arthralgia, hyperthyroidism, pneumonitis, and vitiligo [41,42]. In the phase 2 KEYNOTE-158 study, these more severe pembrolizumab-related side-effects were noted in 12.2% of the patients [37]. Anemia and neutropenia were reported as Grade 3 to Grade 5 events among 30.3% and 12.4% of patients, respectively, receiving pembrolizumab in the phase 3 KEYNOTE-826 trial [40]. 

Nivolumab was assessed as both a monotherapy and as a component of combination therapy alongside ipilimumab to treat HPV-associated cervical cancer in the phase 1/2 CheckMate358 trial. The aim of this trial was to investigate the potential of nivolumab in cases of recurrent and metastatic HPV-associated cervical cancer despite systemic chemotherapy and bevacizumab. In cervical cancer patients, nivolumab monotherapy showed an ORR of 26.3% (95% CI, 9.1–51.2%), with a disease control rate of 68.4% (95% CI, 43.4–87.4%) and a median OS of 21.9 months (95% CI, 15.1 months—not reached [NR]) [43]. Ipilimumab was likely chosen for combination therapy with nivolumab, as it has had promising results when used as an adjunct to radiotherapy in treating various other cancers, including cervical cancer [44]. Two regimens comparing the efficacy of varying nivolumab + ipilimumab concentrations were examined in the study. The first regimen consisted of nivolumab 3 mg/kg Q2W + ipilimumab 1 mg/kg Q6W (regimen A), and the second regimen consisted of nivolumab 1 mg/kg + ipilimumab 3 mg/kg Q3W for four doses, followed by nivolumab 240 mg Q2W (regimen B). How effective each regimen was in patients with or without prior systemic therapy (PST) was also compared, as this factor is prognostic for cervical cancer. In cervical cancer patients without PST, regimen B was found to promote a higher ORR compared to regimen A (46% vs. 32%). This finding was consistent in patients with PST but to a diminished degree of efficacy (36% vs. 23%). Expectedly, PST status appeared to play an immense role in both PFS and OS between the two regimens as well. Patients without PST had a median PFS of 13.8 months (95% CI, 2.1 months—NR) and 8.5 months (95% CI, 3.7 months—NR) for regimens A and B, respectively, while patients with PST had a median PFS of 3.6 months (95% CI, 1.9—5.1 months) and 5.8 months (95% CI, 3.5–17.2 months) for regimens A and B, respectively. Interestingly, the median OS for both regimens in patients without PST was highly successful, with both being NR due to outcomes extending beyond the primary endpoint; while patients with PST had a median OS of 10.3 months (95% CI, 7.9–15.2 months) and 25.4 months (95% CI, 17.5 months—NR) for regimens A and B, respectively [45]. Overall, the higher tolerated dosage of nivolumab + ipilimumab conferred in regimen B appears to promote superior tumor control in cases of metastatic and advanced recurrent cervical cancer to that of regimen A.

The TRAEs of nivolumab monotherapy in patients with cervical cancer were also outlined in the CheckMate358 trial, with the most common presentation being diarrhea (21%). Other TRAEs associated with nivolumab usage include fatigue (15.8%), pneumonitis (10.5%), abdominal pain (10.5%), stomatitis (10.5%), dry eye (10.5%), and arthralgia (10.5%). It appears that nivolumab is well-tolerated, as the aforementioned TRAEs were reported as Grade 1 to 2 in most cases. However, single cases of diarrhea, hepatocellular injury, and pneumonitis were reported as being Grade 3 to 4 [43].

Finally, the phase 3 the EMPOWER-Cervical 1 trial compared the efficacy of cemiplimab to a regimen of investigator’s choice chemotherapy (topotecan or irinotecan) in patients with recurrent or metastatic cervical cancer refractory to first-line platinum-based chemotherapy, regardless of their level of tumor PD-L1 expression [9,46]. There was a significantly longer median OS in the group treated with cemiplimab (12.0 months; 95% CI, 10.3–13.5 months) compared to chemotherapy (8.5 months; 95% CI, 7.5–9.6 months), with an HR of 0.69 (two-sided *p* < 0.001). This significance was maintained when stratifying cases of squamous-cell carcinoma (11.1 months vs. 8.8 months) and adenocarcinoma (13.3 months vs. 7.0 months) [46]. As expected, the level of tumor PD-L1 expression was a determining factor for the response to cemiplimab monotherapy. The highest Kaplan–Meier estimate of OS time was awarded to the patients receiving cemiplimab with a PD-L1 ≥ 1%. There was little variance between patients with PD-L1 ≤ 1% receiving either treatment, with only a minor increase in survival probability from these groups in patients with PD-L1 ≥ 1% receiving chemotherapy [46]. One concern with this study is the variance in the control chemotherapy regimen, as it was left to the discretion of the clinician at the time of administration. While it is unclear how different the findings may have been if a standardized chemotherapy regimen were used, it is clear that the difference between the responses to topotecan and irinotecan were large in this study. In fact, the researchers noted an OS of 6.5 months (95% CI, 4.4–8.8 months) in patients treated with topotecan compared to an OS of 11.8 months (95% CI, 6.9–14.9 months) in patients treated with irinotecan [46].

While generally more tolerable than previously used chemotherapies, complications of immune CPI usage present uniquely as immune-related adverse events (irAEs). Specifically, patients can experience inflammatory or autoimmune responses in previously healthy tissue mediated by off-target cytotoxic T cell activity [47]. For PD-1 and PD-1L inhibitors, these toxicities most commonly present as dermatitis in approximately 17% of patients and endocrinopathies including thyroid and pituitary gland dysfunction, in approximately 10% of patients [47]. Other potential irAEs of these agents include uveitis, pneumonitis, myocarditis, dysphagia, colitis, hepatitis, acute kidney injury, acute interstitial nephritis, encephalitis, myelitis, vasculitis, meningitis, myasthenia gravis, and Guillain–Barré syndrome, inflammatory arthritis, and non-specific symptoms (nausea, vomiting, diarrhea) [47,48]. These potentially life-threatening and otherwise cumbersome side effects generally present over weeks to several months after initial treatment and can present simultaneously in multiple organ systems. Management of these irAEs depends on the severity of the event and typically involves the administration of systemic corticosteroids [47,48].

## 5. Tisotumab Vedotin (Tivdak) Immunotherapy

The innovaTV-204 trial (NCT03438396) was a phase 2 trial exploring the efficacy of Tivdak, an antibody–drug conjugate, as a second-line treatment for recurrent or metastatic cervical cancer with disease progression on doublet therapy [49]. Tivdak is a conjugate molecule consisting of the tissue factor-directed antibody tisotumab and the microtubule inhibitor vedotin. The results of the 101-patient trial showed an objective response rate of 24% (95% CI 16–33) including seven (7%) complete responses and 17 (17%) partial responses, alongside a median duration of response (mDOR) lasting 8.3 months (95% CI; 4.2—NR) [49]. Importantly, the trial showed that Tivdak has a manageable safety profile, with the most common adverse events being alopecia (38%), epistaxis (30%), nausea (27%), conjunctivitis (26%), fatigue (26%), and dry eye (23%) [49]. Given the poor prognosis for recurrent and metastatic cervical cancer, increased duration of response with Tivdak from both innovaTV-201 and innovaTV-204, and confirmation of benefit from an independent review committee assessment of innovaTV-204 using Response Evaluation Criteria in Solid Tumors 1.1, the Food and Drug Administration (FDA) granted Tivdak accelerated approval in late 2021 [49,50]. Follow-up evaluation of Tivdak is currently ongoing, with findings from innovaTV-206, a 2022 Japanese phase 1/2 trial, supporting both the response rate and the duration of effect seen in innovaTV-204 (ORR = 29.2%; mDOR = 7.1 months) [50]. Last, the phase 3 ENGOT-cx12/GOG-3057/innovaTV-301 trial recently began recruitment and seeks to compare the efficacy of Tivdak to investigator’s choice chemotherapy (topotecan, vinorelbine, gemcitabine, irinotecan, or pemetrexed) in patients with second- or third-line recurrent or metastatic cervical cancer [50,51].

## 6. Gemogenovatucel-T (Vigil) Immunotherapy

Vigil immunotherapy is a novel therapeutic vaccine that utilizes patient tumor samples transfected with a plasmid containing a copy of the granulocyte–macrophage colony-stimulating factor (GM-CSF) gene, an immune-stimulatory cytokine, and a bifunctional short hairpin (bi-shRNA) construct that inhibits furin. This therapy inhibits the transfected malignant cells from being able to overexpress transforming growth factor beta (TGF-β), further assisting the patient in being able to mount a CD8+ CTL response against the cancer cells in conjunction with GM-CSF induction [52,53]. This process is particularly important, as cervical cancer is known to overexpress TGF-β, which is immunosuppressive in nature due to its abilities to stimulate angiogenesis and to function as an anti-inflammatory cytokine. The overexpression of TGF-β is a characteristic of malignancies with poor prognoses [54]. Vigil, while not currently tested in patients with cervical cancer, is relevant with respect to activity observed between sequential combination of Vigil and CPI and the standard of care justification of CPI therapy in cervical cancer, as well as benefit demonstrated in ovarian cancer. Vigil combination with two CPIs has been investigated in ovarian cancer. In a phase 1 study of Vigil and atezolizumab patients who received Vigil first vs. atezolizumab first demonstrated improved median OS (NR vs. 10.8 months; HR = 0.33) and reduced Grade 3/4 treatment-related AEs (17.2% vs. 5.1%) [53]. Another study (NCT02725489) investigated the safety and efficacy of Vigil + durvalumab combination therapy in *BRCA*^wt^ patients with ovarian cancer and triple-negative breast cancer. Across all patients, the median PFS was 7.1 months, and the median OS was NR at follow-up of 14.7 months, continuing to support durable OS evidence. Vigil + durvalumab combination therapy was also well tolerated. Overall, 92.3% of TRAEs reported were Grade 1, and 7.7% were reported as Grade 2. There were no Grade ≥ 3 events. The most common adverse event was injection-site reactions (Grade 1) [55]. These studies demonstrate the safety of Vigil in combination with CPI.

Further, *BRCA* mutation increases a woman’s risk of cervical cancer. Vigil has demonstrated efficacy in the *BRCA*^wt^ population in a phase 2b study in patients with newly diagnosed advanced-stage ovarian cancer. Patients with *BRCA*^wt^ molecular profiles were found to have significantly longer RFS after Vigil therapy compared to placebo, with 1-year RFS being 51% (95% CI, 36–71%) in patients treated with Vigil compared to 28% (95% CI, 15–53%) in patients who received the placebo and 2-year RFS of 33% (95% CI, 20–57%) with Vigil compared to 14% (95% CI, 5–39%) in patients who received the placebo. There were no observed survival differences between patients with *BRCA*^mut^ who received Vigil or the placebo [56]. Homologous recombination deficiency (HRD)/homologous recombination proficient (HRP) testing was also performed under blinded assessment, and Vigil demonstrated an RFS HR of 0.382 (90% CI, 0.199–0.750; *p* = 0.007) and OS HR of 0.342 (90% CI, 0.141–0.832; *p* = 0.019 in the HRP patients). Additionally, at three-year follow-up, Vigil-treated patients were found to have an OS of 70% (95% CI, 53.9–91.4%) compared to placebo-treated patients, who had an OS of 40% (95% CI, 23.4–68.4%) (*p* = 0.019, Z-test). This outcome was consistent with data from the time of procurement, when Vigil-treated patients were found to have a median RFS of 18 months (95% CI 14.5 months—NR) compared to placebo-treated patients, who had a median RFS of 12 months (95% CI 11.4–26.1 months) (HR = 0.38; *p* = 0.007) [56,57]. In conclusion, robust data support the role of HRP status as defining a sensitive patient biomarker for Vigil therapy.

## 7. Chimeric Antigen Receptor T-Cells

CAR T-cell immunotherapy is being applied to the development of novel treatments against HPV-associated cervical epithelial cancer. One shortcoming of CAR-T therapy is related to the immunosuppressive activity of tumor-associated macrophages (TAMs), which promote a low-immunostimulatory environment and inhibit CD8+ T-cell function against tumor cells, including engineered CAR T-cells [58]. In a murine model, depleting TAMs with CAR T-cell therapy resulted in an endogenous immune response against previously protected tumors, delaying progression [59]. Thus, additional consideration in targeting TAMs may be necessary to promote effective anti-tumor efficacy in future studies.

A recent phase 1/2 clinical trial utilized CAR T-cells in patients who had tumors positive for HPV16 infection. This study was centered around the delivery of T-cells engineered to have a T-cell receptor (TCR), which were found to have high affinity against the E6_29-38_ epitope of the HPV16 E6 protein [60]. This TCR was derived from the human leukocyte antigen (HLA-A)*02:01 serotype, which the 12 patients selected to participate in this study were confirmed to express. The relative safety of this therapy was also highlighted, as no autoimmune adverse events, off-target toxicities, or cytokine storm was observed [60]. In addition, the most commonly observed adverse event was transient cytopenia, which was reportedly more related to the preparatory treatment than to the immunotherapy itself. However, the results of this study seemingly highlighted a number of issues with this treatment modality more than its efficacy. First, of the 11 participants who were able to have samples collected during a one-month follow-up, two were identified in whom the administered T-cells failed to elicit post-administration recognition of cancer cells expressing HPV E6 epitopes [60]. Additionally, six patients concluded the study with progressive disease, four of whom saw no initial benefit to this treatment modality [60]. Despite this outcome, some positives of this modality were noted. Two of the 12 patients who received E6 TCR T-cells were found to display a partial response to the treatment, with one seeing an approximate 70% reduction in tumor size and the other seeing slightly less than an 80% reduction in tumor size [60]. Additionally, four patients maintained a stable disease state for a period of four months. Nonresponding patients were noted to have either a *IFNGR1* frameshift deletion in their tumor cells or the loss of HLA-A*02:01-E6_29-38_ on the administered T-cells [60]. Another model for resistance against CAR T-cells in these patients could be due to the expression of PD-1 by E6 TCR T-cells and PD-L1 by host tumor-infiltrating immune cells [60]. In addition, there was an inconsistent level of expression of the E6 TCR on administered T-cells. A median of 60% (45–76%) of administered T-cells were confirmed to actually express the receptor, and the composition of administered doses was reported to include medians of 54% CD8+ T-cells (18–79%) and 42% CD4+ T-cells (19–65%) [60].

## 8. Abscopal Effect

Aside from these preventative and therapeutic measures taken to mitigate cervical cancer progression, there have been increasing efforts to elucidate the mechanism underlying the improved outcomes occasionally seen when utilizing immunotherapy treatment concurrently with radiation. A phenomenon known as the abscopal effect has been observed in a number of patient cases in which treatment with immunotherapy resulted in stable disease with an immune checkpoint inhibitor (anti-PD1 therapy), followed by targeted radiation that seemingly results in a change in how tumors respond to the adjuvant treatment systemically—beyond the field of irradiation [61,62].

The mechanism behind the abscopal effect is currently yet fully understood, although an immunostimulatory effect following radiation is thought to be the most likely biological mechanism. Preclinical data have shown significant improvements in systemic immunotherapy efficacy following distal lesion radiation, most notably through enhancement of stem-like CD8+ T-cell expression [63,64]. While preclinical data support the role of CD8+ T-cells role in generating the abscopal effect, consistent results have not been seen clinically. Large clinical trials comparing immunotherapy alone to immunotherapy with radiation have shown statistically insignificant differences, such as the study by McBride et al. [65]. Although the abscopal effect has been reported in 46 cases between 1969 and 2014 and has been well observed in preclinical data, the inability to replicate these preclinical findings within specific subpopulations clinically limit use of the abscopal effect [62,65].

## 9. Conclusions

Overall, the targeted therapies and immunotherapeutic agents being studied and used to treat cervical cancer are robust in both number and mechanism of activity. Promising safety and efficacy profiles have been observed. We expect that, in the coming decade, a number of these modalities will challenge the standard of care and/or be used in combination with conventional agents. Continued clinical examination, clinician oversight, and heightened accessibility measures of these drugs will be critical in advancing cervical cancer management.

## Data Availability

Not applicable.

## Abbreviations

ACOG	American College of Obstetricians and Gynecologists
APC	Antigen-Presenting Cell
BRCA	Breast Cancer Gene
BRCA^mut^	Breast Cancer Gene Mutant
BRCA^wt^	Breast Cancer Gene Wild Type
bi-shRNA	Bifunctional Short Hairpin
CAR	Chimeric Antigen Receptor
Cerclage	Cervical Suture
CPI	Check-Point Inhibitor
CR3	Conserved Region 3
C + P	Cisplatin plus Paclitaxel
C + T	Cisplatin in Combination with Topotecan
DCs	Dendritic Cells
ENTPD1	Ectonucleoside Triphosphate Diphosphohydrolase-1
FDA	Federal Drug Administration
GI	Gastrointestinal
GM-CSF	Granulocyte-Macrophage Colony-Stimulating Factor
GoG	Gynecologic Oncology Group
G + C	Gemcitabine plus Cisplatin
HDR	High Dose Rate
HLA-A	Human Leukocyte Antigen
HPV	Human Papillomavirus
HR	Hazard Ratio
HRD	Homologous Recombination Deficiency
hrHPV	High-Risk HPV
HRP	Homologous Recombination Proficient
IFNs	Interferons
irAE	Immune-Related Adverse Events
LCs	Langerhans Cells
LDR	Low Dose Rate
LVSI	Lymph-Vascular Space Invasion
MCP	Monocyte chemoattractant protein
mDOR	Median Duration of Response
NR	Not Reached
ORR	Operational Readiness Review
OS	Overall Survival
Pap smear	Papanicolaou Test
PD-1	Programmed Death-1
PD-1L	Programmed Death-Ligand 1
PFS	Progression-Free Survival
PST	Prior Systemic Therapy
P + C	Paclitaxel plus Cisplatin
p53	Tumor Protein
QOL	Quality of Life
RFS	Recurrence-Free Survival
RMST	Restricted Mean Survival Time
RT	Radiation Therapy
RTOG	Radiation Therapy Oncology Group
SEER	Surveillance, Epidemiology, and End Results
STI	Sexually Transmitted Infection
TAMs	Tumor-Associated Macrophages
TCR	T-Cell Receptor
TGF-β	Transforming Growth Factor Beta
TLRs	Toll-Like Receptors
TRAEs	Treatment-Related Adverse Events
Tivdak	Tisotumab Vedotin
TPZ	Tirapazamine
T + C	Topotecan plus Cisplatin
US	United States
USPSTF	United States Preventative Services Taskforce
WHO	World Health Organization
WPRT	Whole Pelvis Radiation Therapy
VEGFR	Vascular Endothelial Growth Factor Receptor
VEGF-A	Vascular Endothelial Growth Factor-A
VLP	Virus-Like Particles
V + C	Vinorelbine plus Cisplatin
